# Design and Encapsulation of Immunomodulators onto Gold Nanoparticles in Cancer Immunotherapy

**DOI:** 10.3390/ijms22158037

**Published:** 2021-07-27

**Authors:** Akshita Chauhan, Tabassum Khan, Abdelwahab Omri

**Affiliations:** 1 Department of Quality Assurance, SVKM’s Dr. Bhanuben Nanavati College of Pharmacy, Mumbai 400056, Maharashtra, India; akshitachauhan_97@yahoo.in; 2 Department of Pharmaceutical Chemistry & Quality Assurance, SVKM’s Dr. Bhanuben Nanavati College of Pharmacy, Mumbai 400056, Maharashtra, India; tabassum.khan@bncp.ac.in; 3 The Novel Drug & Vaccine Delivery Systems Facility, Department of Chemistry and Biochemistry, Laurentian University, Sudbury, ON P3E 2C6, Canada

**Keywords:** cancer immunotherapy, immunomodulators, T-cell lymphocytes, gold nanoparticles

## Abstract

The aim of cancer immunotherapy is to reactivate autoimmune responses to combat cancer cells. To stimulate the immune system, immunomodulators, such as adjuvants, cytokines, vaccines, and checkpoint inhibitors, are extensively designed and studied. Immunomodulators have several drawbacks, such as drug instability, limited half-life, rapid drug clearance, and uncontrolled immune responses when used directly in cancer immunotherapy. Several strategies have been used to overcome these limitations. A simple and effective approach is the loading of immunomodulators onto gold-based nanoparticles (GNPs). As gold is highly biocompatible, GNPs can be administered intravenously, which aids in increasing cancer cell permeability and retention time. Various gold nanoplatforms, including nanospheres, nanoshells, nanorods, nanocages, and nanostars have been effectively used in cancer immunotherapy. Gold nanostars (GNS) are one of the most promising GNP platforms because of their unusual star-shaped geometry, which significantly increases light absorption and provides high photon-to-heat conversion efficiency due to the plasmonic effect. As a result, GNPs are a useful vehicle for delivering antigens and adjuvants that support the immune system in killing tumor cells by facilitating or activating cytotoxic T lymphocytes. This review represents recent progress in encapsulating immunomodulators into GNPs for utility in a cancer immunotherapeutic regimen.

## 1. Introduction

Immunological surveillance is a mechanism by which the immune system monitors the body for infections and neoplastically modifies cells to identify alien antigens and kill them with immune cells [1]. It is a concern that active pathogens have evolved a variety of alternative mechanisms to evade immune clearance, by inhibiting phagocytosis and directly destroying immune cells [2]. Cancer cells transform the tumor microenvironment (TME) into a highly immunosuppressive state by activating immunosuppressive immune cells and producing a range of inhibitory checkpoint molecules, cytokines, and enzymes [3]. The effectiveness and intensity of immune responses are hampered by these barriers. Furthermore, abnormal immune cell activation can cause uncontrolled inflammation and lead to allergic, autoimmune, and inflammatory diseases [4]. In addition to transplant rejection, abnormal inflammation can impede tissue and organ regeneration [5].

Immunotherapy is the process of treating a disease by stimulating or manipulating the immune system. Immunostimulatory therapy is used in the treatment of cancer and infectious diseases to activate the immune response, identify and reduce non-self-antigens, and establish memory effects for such diseases [6]. A range of enzymes, cytokines, and immune cells can regulate the immune environment and have been studied to monitor and avoid immune-related disorders. Several immunotherapeutic approaches have shown promising results in the treatment of a variety of diseases. However, low solubility, high immune-mediated toxicity, and bioactivity loss after prolonged circulation may together have a negative impact on the effectiveness of the immunoregulatory agent [7,8].

Nanotechnology is capable of resolving these issues and thus achieve the desirable therapeutic impact. Nanoplatforms have a number of beneficial attributes such as: (a) co-delivery of adjuvants and antigens to the same antigen-presenting cells (APCs) [9]; (b) extension of the half-life of bioactive cargo molecules, thus avoiding enzyme degradation during blood circulation [10]; (c) surface modification improves the drug’s ability to target specific tissues or cells [11]; (d) enhanced tumor deposition in tissues via the enhanced permeability and retention (EPR) effect [12]; (e) increased tolerability of drug doses attributed to less concentration at off-target tissues and organs [13]; (f) stimuli-sensitive actions for secure trafficking and regulated drug release [14,15]; (g) engineered nanoparticles have inherent immunomodulatory properties [16]; (h) diverse in-drug delivery routes that include subcutaneous or intranasal drug administration via a microneedle patch [17,18]; and engineering artificial APCs (aAPCs) for potent T cell activation by surface coupling of both antigens and costimulatory molecules [19].

Nanoparticles have been developed with a variety of physiological and biological activities for drug delivery. Polymer nanoparticles, liposomes, micelles, nanogels, gold nanoparticles, and carbon nanomaterials are some of the commonly studied nano systems. Targeted delivery and stimuli-responsive regulated release of antigens, adjuvants, and immunoregulatory agents have shown phenomenal capabilities in assisting immunostimulatory or immunosuppressive regulation on such nanoplatforms. One technique for enhancing the localization of encapsulated biological compounds in target tissues or cells is to chemically modulate nanoparticles with targeting molecules. For example, nanomaterials coated with DEC-205 antibody (Ab), CD40 Ab, CD11c Ab, or mannose can be selectively internalized by dendritic cells via receptor-mediated endocytosis [20,21,22]. Similarly, CD44, lectins and folate are recognized by associated receptors that are overexpressed on macrophages [23,24,25]. Furthermore, nanostructures made of dextran or dextran sulphate have an inherent targeting feature against macrophages [26]. Recently, nanoparticles with albumin binding domains have been shown to drain towards lymph nodes via a process known as “albumin hitchhiking” [27]. Furthermore, researchers have focused more on the basic functionalization of nanoparticles in the treatment of a broad variety of diseases, in which nanoparticles serve as a primary constituent instead of a delivery platform.

## 2. Strategies Used in Cancer Immunotherapy

The main goal of cancer immunotherapy is to resuscitate the patient’s weakened immune system and enable it to initiate continual strikes on tumor cells, ultimately leading to cancer elimination [28]. According to biological evolution, a tumor cell colony does not utilize all immunosuppressive strategies to survive in a specific host; rather, it utilizes only what is required. As a result, a tumor’s common approach for immune evasion may indicate a possible Achilles’ heel that can be effectively targeted to regain immunological control [29].

**Figure 1:**
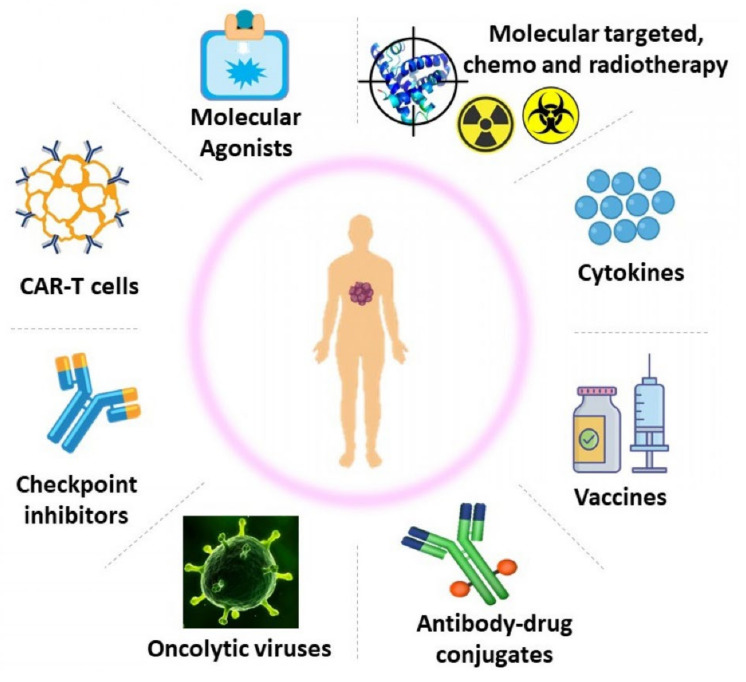
Cancer immunotherapy.

A variety of cancer immunotherapy strategies have been found to be beneficial. Figure 1 depicts the diverse immunotherapies used in the management of cancer [28,30]. A variety of monoclonal antibody drugs have demonstrated substantial anticancer activity in disorders such as malignant melanoma, renal cell carcinoma, Hodgkin’s lymphoma, non–small-cell lung cancer, breast cancer, bladder cancer, and colorectal cancer. Immune checkpoint blockage or immune checkpoint antibody inhibitors prevent immune checkpoint ligands from binding to receptors, allowing T cells to target antigen-expressing tumor cells. Vaccinations are given to patients to develop innate immunity towards current or newly discovered tumor antigens [29]. Several adoptive cell transition experiments have also shown that T cells have a powerful ability to eliminate developing tumors, either directly by CTL activity or indirectly by a variety of CD-4 T cell dependent effector pathways [31]. A number of these strategies activate the immune system in a broad manner, whereas others have a more focused effect. Certain immunotherapies are “customized” via genetic engineering, whereas others, such as monoclonal antibodies, are widely accessible economically [30]. A more in-depth overview of recent cancer immunotherapy techniques is presented here.

### 2.1. Immune Checkpoint Inhibitors

The immuno-suppressive tumor microenvironment (TME) contains upregulated T cells (Tregs), myeloid derived suppressor cells (MDSC), and inhibitory immunological checkpoints that frequently compromise the therapeutic potential of adoptive T cells in patients [32]. Both inhibitory and stimulatory surface receptors are expressed by activated T cells. The most researched inhibitory proteins are Programmed death ligand 1 (PD1) and cytotoxic T-lymphocyte associated protein 4 (CTLA4). When these receptors bind to their ligands, they transmit downstream signals that block T cell activation. These immunological check points provide protection to the host from excessive T cell activation [33]. Tumor cells, in contrast, use this strategy to avoid being eliminated by overexpressing inhibitory receptor ligands. Suppressing inhibitory receptor–ligand interactions can significantly improve T cell mediated tumor treatment [34,35]. Table 1 indicates the FDA-approved immune checkpoint inhibitors. Therefore, immune checkpoint inhibitors (ICI) provide a significant advancement in cancer therapy.

#### 2.1.1. PD-1/PD-L1 Blockade

**Figure 2:**
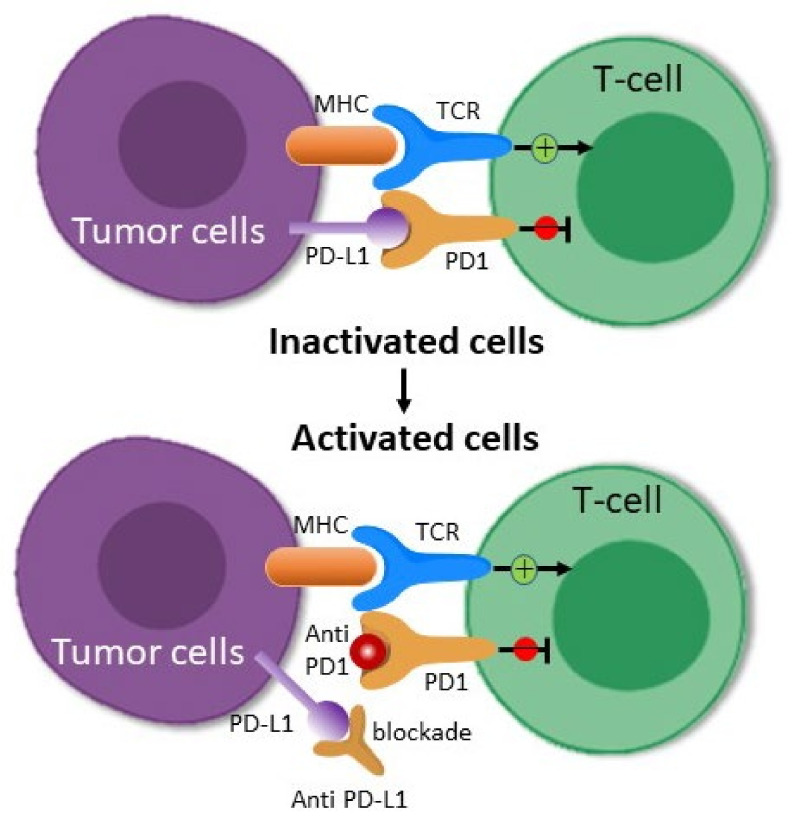
Anticancer mechanism of PD-1/PD-L1 inhibitors. Tumor cells escape from the anti-tumor activity of T cells by the binding of PD-L1 to the PD-1 receptor. PD-1 or PD-L1 antibodies block the binding of PD-L1 on tumor cells to PD-1 receptors on T cells, which allows T cells to target tumors.

The PD-1 receptor is present on active CD8+ T cells, and when it interacts with its PDL-1 or PDL-2 ligands, it inhibits T cell activation [36]. PDL-1 receptors are continuously expressed on the cancer cells’ surface via poorly recognized oncogenic signaling pathways, or they can also be released in response to immune-stimulating cytokines such as interferon-α [37]. PDL-1 expression in response to cytokines is referred to as “adaptive immune resistance”. An antibody that attaches to either PD-1 or PDL-1 disrupts the inhibitory connection and restores T cells’ ability to target tumors (Figure 2) [38]. Clinical trials have shown that PD-1/PDL-1 inhibitors are beneficial in treating melanoma, Hodgkin disease, NSCLC, and bladder cancer [39].

Lipson et al. revealed the outcomes of a phase I trial in three patients with melanoma, renal cell cancer, and colorectal cancer who were medicated with BMS-936558 (PD-1 antibody); they showed partial response to melanoma and a complete response to renal and colon cell cancers. In addition, after melanoma remission, reinduction therapy was found to be successful [40]. In another study, Lipson et al. examined the clinical effectiveness of pembrolizumab (aPD-1) in a patient suffering from metastatic PD-L1 basal cell carcinoma [41]. Similarly, a phase III double-blinded clinical study evaluated pembrolizumab as an adjuvant therapy in melanoma. After complete resection, it was observed that patients who received pembrolizumab every three weeks for up to one year had significantly longer recurrence-free survival than those who received placebo (NCT02362594) [42]. A further clinical trial confirmed aPD-1 activity in two out of ten patients with enzalutamide-resistant prostate cancer [43]. New research has shown that toxicity can be controlled in a combination strategy, and numerous inhibitors are progressing towards large scale studies or acquisition of FDA approval for cancer therapy [44]. However, checkpoint treatments are most successful when PD-L1 is represented in immune cells (macrophages and T cells) and tumor cells, thus allowing customization of treatment and results.

#### 2.1.2. CTLA-4 Blockade

CTLA-4 is a major inhibitory receptor highly expressed by CD4+, CD8+, memory, and regulatory T cells. It fights for CD80 and CD86 binding on APCs with CD28, a key costimulatory receptor necessary for T cell activation. When these ligands bind to CTLA-4, T cell proliferation and cytokine production are reduced [45]. Antibodies against CTLA-4 have been produced to combat the effects of inhibitory immune regulators [46]. Ipilimumab (anti-CTLA-4) was the first checkpoint antibody authorized by the FDA to treat melanoma [47].

CTLA-4 blockade has been used in clinical studies as an immunomodulatory therapy because various cancer cell lines, such as small cell lung cancers (NCT03043872), non-small cell lung tumors (NCT02352948, NCT02453282, NCT02542293, NCT03164616), hepatocellular carcinoma (NCT02519348), and gastric adenocarcinoma (NCT02340975), inhibit immune stimulation and cytotoxicity [48]. Studies have also revealed remarkable advantages in pancreatic and lung cancer treatments, particularly when combined with other medicines [49].

Nivolumab (aPD-1) in combination with ipilimumab (humanized aCTLA-4) is now being studied in over 100 clinical studies at varying phases [50]. The combination of aCTLA-4 and aPD-1 has a synergistic effect on tumor-induced lymphocyte (TIL) activity while decreasing the frequency and function markers of Treg cells [51]. An advantage of this strategy is to lower the immune-related adverse effects in patients. For example, decreased ipilimumab dose in 153 melanoma patients followed by standard pembrolizumab (aPD1) was observed to offer significant anti-tumor immune responses and lower toxicities [52]. As a result, existing clinical evidence suggests that checkpoints combination regimens are expected to improve results against a wide range of tumor types.

### 2.2. Adoptive T Cell Therapy

One fundamental concept of cancer immunotherapy is to elicit a significant and long-lasting T cell response [53]. Adoptive T cell treatment is a type of passive immunization that involves the transfusion of T cells that are being produced or altered in vitro [54]. In this therapy, a patient’s white blood cells are collected (leukapheresis), and T cells that recognize tumor antigens are separated and cultivated in vitro in massive quantities, triggered by cytokines, or genetically altered before being re-infused into the patient as “live medicines” to combat cancer [55]. Adoptive T cell treatment has several drawbacks, including the fact that it takes time, is quite expensive, and requires laboratory skill. CTL therapy, TIL therapy, and engineered T cell-based therapy are some of the existing applications of adoptive T cell therapy [56].

#### 2.2.1. Cytotoxic T-Lymphocyte Therapy

CTL therapy is based on the in vitro activation of pure CD8+ T lymphocytes with a tumor-specific cytokine and antigen. The long-term growth of T cells requires cytokines, especially IL-2. The main obstacle for CTL development is a shortage of stable APCs.

#### 2.2.2. Tumor Infiltrating Lymphocytes Therapy

TILs are T cells that are located inside a tumor. T cells are isolated and enriched from resected tumors that can respond to antigens present in tumors. The stage of choosing single T cells from a patient’s blood is eliminated with this method. It has demonstrated promising results in melanoma treatment, but not in other malignancies [57].

#### 2.2.3. Genetically Engineered T Cells

Exogenous production of receptors that identifies tumor antigens on the T cell surface prior to reinfusion into the host is now possible because of advances in genetic technology. These changes enable T cells to precisely target tumor antigens and stimulate T cell activation upon antigen binding. T cell receptors (TCRs) and chimeric antigen receptors (CARs) are the two main types of receptors that can route T cells towards the tumor. Peripheral blood T cells can produce both TCR-T and CAR-T cells.

TCR treatment is constrained to major histocompatibility complex (MHC)-compatible patients because antigens are identified by TCR-T cells in the presence of MHC. By comparison, MHC expression is generally downregulated in tumors, and antigens for TCR binding are lost. To resolve these difficulties, chimeric antigen receptors (CARs) were created [58,59].

CAR-T cells are created by genetically modifying an antigen-binding cell surface, which is typically a single-chain variable fragment antibody (scFv), and transforming them into cytotoxic T lymphocytes. When antigens bind to T cells, downstream signaling pathways are triggered, which promotes T cell proliferation, cytokine release, and tumor cell eradication. CAR-T cells may identify cell surface antigens in a non-MHC constrained form and tumor variations with minimal MHC expression do not compromise them [60]. A single CAR design may potentially be utilized to treat all tumors that express the same antigen.

### 2.3. Immunological Adjuvants

Immunologic adjuvants are chemicals that, when combined with particular vaccine antigens, increase the strength, onset, or duration of antigen-specific immune responses. Adjuvants are often non-antigenic when administered in the absence of vaccine antigens. Traditionally, just one form of adjuvant, i.e., aluminum salts, was approved for human use. Inorganic substances such as aluminum hydroxide and aluminum phosphate facilitate aggregation and physical deposition effects for complexed antigens. Inflammatory reactions are triggered by such adjuvants, which are occasionally regulated via Toll-like receptors (TLRs) and other related molecules [61]. Various immune receptors such as TLRs expressed on the membranes of dendritic cells, NK cells, macrophages, and cells of adaptive immunity can be activated by adjuvants that mimic damage or pathogen-associated molecular patterns [62].

Numerous clinical studies have shown that immunostimulatory adjuvants, particularly TLR agonists, have therapeutic benefits, not just as vaccine adjuvants, but also as anticancer therapy [63,64]. However, precise adjuvant delivery to APCs is still a concern. To circumvent this constraint, both natural and synthetic developed nanoparticles have been extensively used. Several cancer vaccines in human trials comprise lipid-based nanoparticles made with already authorized molecules and given in combination with immunostimulatory adjuvants [63,64].

### 2.4. Cancer Vaccine as Immunotherapy

Vaccinations is an effective immunization method to create false immunity against a particular pathogen to avoid future infections [65]. A cancer vaccination stimulates the body’s own immune system to prevent or treat cancer [66]. Vaccines are substantially affordable and easier to administer than adoptive T cell treatments. Cancer vaccines have been developed to selectively target tumor-specific antigens, in comparison to anti-blockade therapy, which does not release T killer cells [67]. As a result, the off-target consequences are properly managed. Because of these benefits, cancer vaccines have recently received a significant amount of interest [68]. In mouse fibrosarcoma, dendritic cells generated from GM-CSF served as both antigen-presenting cells and antigen donors [69]. Studies also revealed that combining photodynamic therapy (to boost antigen-presenting ability) with a dendritic cell vaccine can generate an efficient immune response in squamous cell carcinoma patients [70]. An adenoviral vector was utilized in another combination therapy to specifically stimulate dendritic cells [71]. Although huge progress has been made in normal vaccines, few vaccines have been approved for cancer due to their inefficiency in clinical studies [72].

## 3. Gold Nanoparticles

Gold nanoparticles (GNPs) are currently being extensively explored due to their enormous prospects in nanotechnology, particularly bio-nanotechnology, for detection, imaging, and treatment [73]. Metallic NPs have a higher surface area to volume ratio than analogous bulk materials, thus providing higher binding affinity, large surface energies, unique electronic structures, and the possibility for plasmon excitation [74]. GNPs have all of these features and are regarded as one of the most stable metallic NPs. Colloidal gold has a wide range of uses in the treatment of a wide variety of diseases, particularly due to its optical and magnetic properties. These properties have demonstrated reduced cytotoxicity and the ability to interact with numerous ligands and functional groups that have a high affinity towards the gold surface, which facilitates interactions with other biomolecules [75]. The use of GNPs in clinical studies has grown because of their facile synthesis [76] and ease of processing to achieve high-quality, high-yielding, and size-controlled colloidal gold. Gold has a broad range of applications due to the various compositions and configurations available, such as NPs, nanocages, nanoshells, nanostars, and nanorods [77].

The anticancer mechanisms of encapsulated metallic NPs are still unknown. Table 2 depicts the benefits of encapsulating compounds versus no-encapsulated compounds in terms of drug release, distribution, accumulation and associated side effects. Nonetheless, data has revealed that activation of p53 protein and caspase-3 expression, suppression of VEGF-induced activities, reactive oxygen species (ROS) formation, and sub-G1 cell arrest are the major possible anti-cancer mechanisms related to metallic NPs [78]. Metallic NPs cause DNA damage via oxidative DNA damage, mutation, DNA strand breaks, chromosomal abnormalities [79], and oxidation of DNA bases due to excessive formation of reactive oxygen species (ROS) [80]. Moreover, metallic NPs may cause the release of cytokines such as IL-1, IL-6, and TNF-α, which can cause DNA damage and inhibit DNA repair [81]. The cellular antioxidant defense system is defeated by the overproduction of ROS within the cell [81]. Glutathione is an important component of the cellular antioxidant defense mechanism. Specifically, glutathione is a tripeptide comprising glutamic acid, glycine, and cysteine that plays a vital role in cells, such as regulating cell proliferation and apoptosis, direct chemical neutralization of singlet oxygen, hydroxyl radicals, and superoxide radicals. Metallic NPs may also inhibit the VEGF-induced signaling pathway [82]. VEGF stimulates the activation of the VEGF receptor-2 (KDR/Flk-1) enzyme cascade, which controls cell survival, migration, proliferation, and vasodilation [82].

The key processes involved in the biological response of cells to GNPs are somewhat similar to those of metallic NPs, such as the formation of ROS and oxidative stress, DNA damage induction, cell cycle impacts, and potential interference with bystander effects [83]. The combination of the NP surface with O_2_ is a process that has been identified as a probable cause of cytotoxicity. Donor electrons are transported from the surface of the NPs to oxygen molecules during this process, resulting in superoxide, which can lead to ROS generation via dismutation [84]. Another analysis revealed that mitochondria appear to be implicated, with tests demonstrating function loss due to high intracellular ROS levels [85]. ROS can oxidize the mitochondrial membrane, disturbing its potential and releasing additional superoxide anions into the cytosol, which can then be transformed into H_2_O_2_ molecules. These then spread across membranes, causing DNA damage [85]. This is confirmed by experimental observations utilizing 1.4-nm triphenyl monosulfonate (TPPMS)-coated GNPs that induce mitochondrial potential loss via enhanced oxidative stress, resulting in necrotic cell death [86]. Furthermore, antioxidants with thiol groups were discovered to bind to the surface of GNPs. This indicates that GNPs can attach to these antioxidants inside cells, preventing endogenous reducing agents from working and thereby lowering the cell’s redox capacity [86].

As a prospective medicine option, GNPs do not alter cell viability and gold medicine has a strong record of utilization in treatments of epilepsy, arthritis and rheumatic disorders [87]. Based on this, one can assume that GNPs may interact with metabolic processes while also conserving cell viability. Biosafety was a major concern in previous research of GNPs in therapeutic systems [88]. For instance, Ravi Shukla et al. monitored the entry and biodistribution of fluorescein isothiocyanate (FITC)-conjugated GNPs within macrophages. Encapsulated GNPs do not result in cytokine excretion, but lower the generation of cellular reactive oxygen species [89]. Surprisingly, other papers have described a similar outcome when chloroauric acid [90] or cyanobacterium were added to cell cultures [91]. GNPs can be synthesized by both eukaryotic and prokaryotic organisms, implying that GNPs may interact with cellular metabolism. GNPs have also been studied in relation to metabolic processes. Li et al. investigated that insertion of 20 nm GNPs to human lung cancer cells (MRC-5) increased oxidative stress and autophagy, thus resulting in genomic instability [92]. Ma et al. utilized a similar method to show that increased autophagy was caused by excessive autophagosomes because of lysosome impairment and GNP uptake [93]. Furthermore, the lysosome was not the only organelle that was altered by intracellular NP ingestion. Wang et al. demonstrated that gold nanorods (GNRs) mainly accumulate within the mitochondria [94]. When murine melanoma cell line (B16F10) cells were treated with 13 nm GNPs for 20 h, NPs accumulated throughout the endoplasmic reticulum and Golgi apparatus [95]. The deposition of GNPs within specific cellular areas may disrupt normal cellular metabolism, but not cell viability. Wang et al. showed that dietary feeding of GNPs to Drosophila larvae resulted in activation of the PI3K/Akt/mTOR signaling pathway and substantial lipid deposition compared to controls [96]. It is well understood that abnormal PI3K signaling can alter metabolism and cause type 2 diabetes and tumorigenesis [88]. As a result, prior to clinical application, an optimization model of a functional GNP is required.

## 4. Biodistribution and Immune Response of GNPs

The blood clearance and organ accumulation of GNPs in vivo is affected by various factors such as particle size, shape, charge, and coating [97]. However, GNPs unavoidably accumulate in high concentrations in the liver and spleen, making it critical to understand how GNPs interact with the immune system. Although research on the immunological effects of gold nanoparticles is still in its early stages, a number of in vitro and in vivo results are notable [98]. Yen et al. investigated the effects of GNPs on macrophages in vitro and discovered that GNPs can stimulate pro-inflammatory cytokine expression in a size-dependent manner [99]. Furthermore, some studies have identified that GNPs can block IL-1-mediated inflammatory responses [100] and Toll-like receptor 9 (TLR-9) responses [101] in a size-dependent manner.

Tsai et al. discovered that 13 nm GNPs bind to the immunosuppressive TGF-β1 protein released by murine bladder tumor 2 cells (MBT-2) in a time- and dose-dependent manner [102]. The cytokine’s adsorption to the nanoparticle surface also resulted in a conformational shift that inhibited biological activity of TGF-β1, resulting in a reduction in the epithelial-mesenchymal transition of murine mammary gland cells in vitro. Finally, the evidence suggests that when MBT-2 cells were implanted in mice in the presence of GNPs, tumor growth was inhibited. The particles were also found to diminish the systemic concentration of TGF-1 and the concentration of the immune suppressive cytokine IL-10. Furthermore, tumors implanted with GNPs demonstrated increased infiltration of CD4+ and CD8+ T cells. Collectively, the findings show that GNPs can inhibit TGF-β1 signaling and thereby stimulate an anti-tumor immune response [102]. More research into the interactions of GNPs with other immune regulating cytokines could lead to novel immunological therapeutic applications for GNPs.

## 5. GNPs in Cancer Immunotherapy

**Figure 3:**
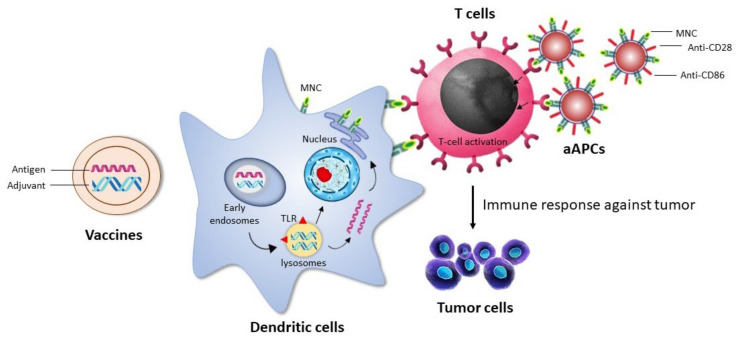
Nanoparticle-based delivery system to activate dendritic cells or T cells for immunotherapy.

Immunotherapy is presently regarded as one of the most efficient strategies for cancer treatments [103]. The application of nanomaterials in the distribution of immunotherapeutic drugs has been widely investigated and is regarded as an interesting field of study. Immunotherapeutic nanoparticles have become a viable approach in cancer therapy due to their higher selectivity, potency, visualization ability, and therapeutic efficacy [104,105,106,107,108,109,110,111,112]. In addition to the targeted anti-cancer drug delivery, GNPs are a desirable nanoparticle for cancer immunotherapy for multiple purposes [113,114]. GNPs enable imaging and photothermal possibilities due to their plasmonic property, which can be utilized with various treatments to track the progression of an immune system response. GNPs are suitable for numerous adjuvants and antigen delivery, which helps the immune system to kill cancerous cells by boosting or triggering cytotoxic T cell lymphocytes due to their well-developed surface chemistry. Thus, GNPs are utilized to build artificial APCs, which may functionalize with co-stimulating chemicals and MHS-complex proteins packed with antigen peptides. Ultimately, GNPs can operate as adjuvant NPs by binding with dendritic cells and activating cytokine production (Figure 3) [115]. There are no reports of immunomodulator-based GNPs in the market but many are in clinical trials.

## 6. Role of GNPs in Drug Delivery

The unique features of GNPs have traditionally been recognized as promising aids for cancer diagnostics and drug carrier purposes. These features include large surface area-to-volume ratio, high plasmonic resonance, morphology characteristics, multi-functionalization, ease of production, and stability [116]. Several medications can be immobilized on the GNP’s surface, mainly via direct -S or -N binding, ligand bonding, hydrogen bonding, van der Waals forces, and electrostatic interaction. Generally, -N binding provides more potential for drug delivery in cancer cells than -S binding because there is stronger interaction between S and Au [117]. Furthermore, GNPs are non-toxic, non-immunogenic, and have excellent retention and permeability. They also provide additional benefits by allowing drugs to rapidly permeate and deposit at tumor areas [118]. GNPs are being used to develop a variety of novel techniques. Table 3 depicts the advantages and limitations of GNPs in the therapeutic management of cancer. Notably, newer approaches to enhanced delivery systems not only target immune and cancer cells, but also enhance the richness of immuno-therapeutics within lesions [103]. Innovative immunotherapy delivery techniques are being explored and designed, such as those including nanoparticles, hydrogels, and scaffolds [119], and GNPs offer a highly attractive carrier system for this application.

### 6.1. GNPs Based Immune Adjuvants

The approved PD-1/PD-L1 medications (atezolizumab, avelumab, cemiplimab, durvalumab, nivolumab, and pembrolizumab) are being successfully used to enhance the survival of cancer patients in combination with chemotherapy and targeted drug delivery by inhibiting binding of PD-1 to PD-L1 and preventing escape of cancer cells from the immune system via the use of antibody-based drugs [120].

Emami et al. synthesized doxorubicin (DOX)-conjugated anti-PD-L1 targeted gold nanoparticles (PD-L1-GNPs-DOX) for colorectal cancer. Because of their drug resistance property, DOX and PD-L1 antibodies are difficult to administer to tumor areas due to the TME barrier. As a result, a PD-L1-AuNP-DOX hybrid was effectively developed and enabled the intracellular absorption of DOX in CT-26 cells, as demonstrated by strong apoptotic effects (66%). The combination of PD-L1-AuNP-DOX therapy and NIR irradiation inhibited CT-26 cell proliferation in vitro by enhancing apoptosis and cell cycle arrest [121,122]. Furthermore, utilizing GNPs to distribute PD-1/PD-L1 antibodies or siRNA is an excellent approach to block PD-1 tumor immunological checkpoints. Meir et al. utilized the targeted drug release strategy and, hence, modified the PD-L1 antibody onto the GNP’s surface. Studies in a colon cancer mouse model revealed that the combination of computed tomography imaging and GNPs conjugated to α-PD-L1 enabled prediction of therapy response within 48 h of treatment. This was obtained using noninvasive measurements of NP accumulation levels within tumors, which revealed that the NPs effectively stopped tumor growth with only one-fifth of the standard treatment dosage [123].

Liu et al. described a new nanoplatform of GNPs@PSS/PDADMAC-siRNA (GNPs-siRNA) that was designed and synthesized by coating GNPs with poly(sodium 4-styrenesulfonate) (PSS) and poly(diallyldimethylammonium chloride) (PDADMAC) to transport small interfering RNA (siRNA). However, PD-L1 is crucial for cancer cell survival through the intrinsic signaling activities, in addition to serving as an important checkpoint gene in the immune system. Hence, Liu et al. successfully attached the human PD-L1 siRNA to the surface of GNPs@PSS/PDADMAC to obtain the GNPs-hPD-L1 siRNA nanoplatform. In vitro and in vivo studies confirmed that the GNPs-hPD-L1 siRNA not only served as a carrier for siRNA delivery to downregulate hPD-L1 expression, but also as a photoacoustic imaging agent and photothermal agent for photothermal therapy in human lung cancer cells [124].

Merino et al. designed DOX immunoliposomes with functionalized monovalent-variable fragments of α-PD-L1. These immunoliposomes were tested in vitro and in vivo in a B16-OVA melanoma murine cell line that over-expressed PD-L1. The analysis indicated that immunoliposomes bound specifically to PD-L1+ cells, leading to higher cell interaction and Dox internalization, and lowering the IC50 up to 30-fold when compared to conventional liposomes [125]. The findings revealed that the interaction of the PD-L1 antibody with GNPs and doxorubicin could not only enhance the apoptosis of cancer cells, but also inhibit tumor cell-mediated angiogenesis.

### 6.2. GNPs Based Genetic Drugs

GNPs can be exploited as potent nano-materials in siRNA delivery for two purposes: (a) the fabrication of multi-functional monolayers is a simple means to achieve a variety. (b) due to their low toxicity, there is small size difference and selective gene transfection. At present, GNPs are one of the most widely used carriers for siRNA as an anticancer therapy [126].

Hou et al. developed non-viral pDNA/siRNA delivery vectors, i.e., dendrimer-entrapped gold nanoparticles (G-DENPs) that were substantially altered with polyethylene glycol monomethyl ether. Encapsulated G-DENPs were used to transport B-cell lymphoma-2 (Bcl-2) siRNA to human cervical cancer cell lines, thereby inhibiting the increased green fluorescent protein and luciferase reporter genes. However, PEGylated G-DENPs could hold considerable potential for usage in pDNA and siRNA delivery applications due to improved pDNA/siRNA transfection efficiency and lower cytotoxicity. [127].

Labala et al. designed layer-by-layer integrated GNPs (LbL-GNPs) containing anti-STAT3 siRNA and imatinib mesylate (IM) for the treatment of melanoma. This strategy revealed stronger suppression of the STAT3 protein, lower cell viability, and enhanced apoptotic activity compared to LbL-GNPs carrying STAT3 siRNA or IM. In vivo efficacy trials in melanoma tumor-bearing mice revealed that non-invasive topical iontophoretic delivery of LbL-GNP (0.5 mA/cm^2^) was equivalent to that of intra-tumoral administration. When STAT3 siRNA and IM were delivered together via LbL-GNP, there was a significant reduction in percentage tumor volume, tumor weight, and reduced STAT3 protein expression compared to either STAT3 siRNA or IM-packed LbL-GNP [128]. In conclusion, integrating GNPs with the RNAi route to transport siRNA and small molecules (imatinib mesylate) is one method of developing a drug delivery system.

Xue et al. developed a functional nanoplatform based on generation 5 (G5) poly(amidoamine) (PAMAM) dendrimer-entrapped gold nanoparticles (G-DENPs) as a nonviral vector for delivering PD-L1 small interfering RNA (siPD-L1) for subsequent PD-L1 gene silencing-mediated tumor immunotherapy. The functional Au DENPs developed had adequate water dispersibility, colloidal stability, and good cytocompatibility following complexation with siPD-L1, in addition to efficient gene delivery performance. However, studies revealed that functionalized G-DENPs may carry a programmed siRNA-PD-L1 (siPDL1) to cancer cells, efficiently downregulate PD-L1 protein expression, and boost CD8+ and CD4+ T cell infiltration in spleen and cancerous tissue, thereby facilitating immunotherapy. Because it has a much higher tumor suppression efficacy compared to the PD-L1 antibody [129], the siRNA-GNP delivery system could provide a significant opportunity in immunotherapy.

### 6.3. GNPs Based Tumor Vaccines

GNPs are identified using non-invasive imaging technologies, offering physicians data about vaccination delivery that aid in the analysis of therapeutic efficacy [130]. Cao et al. developed a paradigm of a hyaluronic acid (HA) and ovalbumin (OVA)-entrapped GNP-based (HA-OVA-GNP) vaccine for photothermally regulated cytosolic antigen delivery with near-infrared (NIR) irradiation, which was found to trigger antigen-specific CD8+ T cell responses. The chemical interaction of thiolated HA and OVA with AuNPs promotes dendritic cell antigen absorption via receptor-mediated endocytosis. HA-OVA-GNPs promote antigen absorption via receptor-mediated endocytosis, which improves thermal energy translation and NIR absorption. As a result, under laser irradiation, the HA-OVA-GNP nanovaccine can efficiently activate a strong anticancer immune system response [131].

In another investigation, Shinchi et al. designed a paradigm of GNPs mobilized with α-mannose as a carrier for a TLR-7 ligand that activates immune cells. The synthetic TLR7 ligands, i.e., 2-methoxyethoxy-8-oxo-9-(4-carboxy benzyl) adenine (1V209) and α-mannose were co-immobilized on the GNP surface using linker groups made up of thioctic acid (1V209-αMan-GNPs). The 1V209-αMan-GNPs demonstrated stronger extracorporeal cytokine generation ability than the unconjugated 1V209 derivative in mice bone marrow-derived dendritic cells and human peripheral blood mononuclear cells. In immunized research, 1V209-αMan-GNPs generated noticeably greater titers of IgG2c antibody particular to ovalbumin than an unconjugated 1V209, with no evidence of weight loss or splenomegaly. These findings indicate that 1V209-αMan-GNPs may be effective and safe adjuvants for the formulation of cancer vaccines [132]. Cancer immunotherapy highly depends on GNPs, which act as carriers for tumor vaccines. However, GNP-based vaccines are innovative and effective anticancer therapies.

## 7. Combinatorial Effects of GNPs in Cancer Immunotherapy

The utilization of GNPs coupled to CpG oligo-deoxynucleotides (CpG-ODNs) is among the most prominent variations. These native CpG-ODNs are unable to pass the cytoplasm via the cell membrane and are rapidly destroyed by cell cytoplasm nucleases. The conjugation of ODN–GNP exhibits high potency in vitro and is utilized for intracellular administration [133,134,135]. When GNPs are crosslinked with CpG-ODNs, the efficiency of Au nanospheres with a diameter of 15–50 nm exceeds that of nanoshells, nanorods, and nanostars [130]. In addition to CpG-ODNs, cancer immunotherapy has used GNPs in combination with TNF-α, TGF-β, the PDL1 inhibitor and TLR-7 agonist, specialized antibodies, and other tumor cell death factors or/and immunostimulants [123,136,137,138,139,140]. GNPs exhibited a considerable proclivity to associate with dendritic cells [141], causing the release of immunostimulatory cytokines (TNF-α, IL-1, IL-6 and IL-12) [142,143] and the down-regulation of immunosuppressive chemokines (TGF-β1 and IL-10) [102].

GNP-based nanovaccines (GNVs) are being designed on the surface of tumor peptide antigens with a higher density, and for which each GNP has the capacity to carry 1300 peptides [144]. The suggested vaccines successfully distribute peptide antigens to dendritic cells, where they bind with MHC-I and initiate responses of the anti-tumor immune system [144]. Lin et al. employed a self-assembling conjugation approach to create GNVs, which were then tested via DC-to-splenocyte interferon-γ enzyme-linked immunosorbent spot tests. This GNV formulation has demonstrated successful peptide conjugation with a yield of around 90% while remaining less than 80 nm in diameter. DCs ingest GNVs with low toxicity and can process the vaccine peptides on the particles to trigger cytotoxic T cells (CTLs). These GNVs with high peptide density activate CTLs faster than free peptides and represent a promising approach as vaccine carriers for a variety of vaccine types. [145]. A high-density peptide on the GNP surface can trigger cytotoxic T cells more effectively than free peptides, suggesting that GNPs have tremendous promise as vaccine carriers. Researchers of nanovaccines have utilized a variety of tumor antigens of both carbohydrate and protein [146,147].

Adjuvant effects are also provided by GNPs. Ahn et al. developed a GNP-based antigen delivery system capable of generating efficient humoral and cellular immunity against an endogenous tumor-associated self-antigen, which could possibly serve as a cancer vaccine without the use of a particular adjuvant. Interestingly, they found that AuNP/EDB-OVA 257–269 successfully promoted antigen cross-presentation in professional DCs, resulting in antigen-specific CTL responses. The vaccine efficiently targeted local LN targets after injection of AuNP/EDB-OVA 257–269, leading to a high degree of EDB-specific antibody production and, eventually, suppressing tumor growth in an EDB-overexpressing breast tumor model [148].

Delivering antigens to the lymph nodes is a suitable approach because the lymph node contains a large number of dendritic cells, immune cells, and macrophages that are essential for developing cellular and humoral immunity [149,150]. As a result, nanoparticles are used to deliver antigens to lymph nodes in a size-dependent manner via lymphatic channels [151]. Several preclinical investigations have shown that GNPs can be used to treat cancer immunotherapy [152]. The utilization of stimulated dendritic cells has emerged as a new technique in cancer vaccine production, and GNPs of varying size, shape, and structure serve as carriers for this kind of therapeutic vaccine [153]. However, GNPs affect cellular uptake, dendritic cell maturation, T cell differentiation, and cytotoxicity in human dendritic cells [154]. Dendritic cell maturation and lymphocyte proliferation were also enhanced by GNPs loaded with tumor antigens [155]. Adjuvant activity on dendritic cells or GNP-induced dendritic cell tuning occurs when effective vaccinations relying on dendritic cells and GNPs may induce phagocytosis, migration, maturation, T cell co-stimulation, and cytokine release [156].

Cruz et al. synthesized three new peptides with several modifications at the N-terminal (palmitoyl, acetyl, and FITC). To allow grafting onto GNPs, these peptides had a Cys as a C-terminal residue. The results showed absorption and stimulation of immunological responses with 13 nm GNPs coupled with prostate cancer peptide antigens via dendritic cells. GNPs and liposomes targeting Fc receptors of human DCs, in contrast, are excellent antigen delivery carriers that elicit a significant immune response when compared to non-targeted luteinizing hormone-releasing hormone–nanoparticle conjugates, and a superior response when compared to naked antigens [157]. GNPs conjugated with IgG peptides and Fc fragments were found to engage with dendritic cell Fcg receptors and then diffusely locate in the cytoplasm after uptake. Internalization of antigen-conjugated GNPs in dendritic cells boosted immunological response, whereas using the original antigen only stimulated lymphocyte proliferation. According to Cruz et al., this strategy provides an opportunity for the development of an effective method for producing anti-cancer and other vaccines.

Dreaden et al. proposed utilizing GNPs coupled with macrolide antibiotics (tricyclic ketolide, clarithromycin, and azithromycin), that can aggregate in tumor-specific macrophages and promote cytotoxicity, leading to cancer cell death. Cardioid immersion darkfield scattering microscopy was used to examine the preferential uptake/accumulation of macrolide–GNRs into tumor-associated macrophage (TAM) cells. TAM cells (RAW 264.7) displayed much higher levels of macrolide–GNR uptake than either squamous cell carcinoma or non-malignant keratinocyte cells, whereas PEG–GNRs showed only nominal cell surface binding. However, the data reveal that GNP-activated macrophages may boost TAMs’ innate cytotoxic responses to the malignancies they infiltrate. Furthermore, macrolide–GNRs that actively target TAMs can improve anti-tumor response and achieve higher selective delivery due to the size-dependent enhanced permeability and retention (EPR) effect [158].

Wang et al. synthesized bovine serum albumin (BSA)-coated gold nanorods (BSA-coated GNRs), which they used for photothermal ablation of breast tumor cells. The BSA-coated GNRs had a high photothermal conversion efficiency and a good photothermal ablation effect on tumor cells. The ablated tumor cells were co-cultured with immature dendritic cells (DCs) using a direct cell contacting model and a diffusion model to verify the stimulatory effects of cell–cell interaction and soluble factors secreted by ablated tumor cells. The breast tumor cells were efficiently ablated by the up-taken BSA-coated GNRs after NIR laser irradiation when the gold concentration in the cell culture medium was 0.4 and 0.6 mM. At a low Au concentration of 0.2 mM, the BSA-coated GNRs could partially ablate the breast tumor cells. As a result, the outcomes showed that BSA-coated GNRs have a remarkable photothermal converting efficiency, in addition to a powerful photothermal ablated impact on tumor cells. [159].

Choi et al. also proposed a new approach for photothermal therapy of malignancies. This approach exploits a “Trojan horse” in the form of monocytes and macrophages loaded with phagocytosed GNSs, and was inspired by the findings on GNP uptake via macrophages [160].

## 8. Conclusions

Advances in chemical synthesis of GNPs has resulted in a wide range of particles with variable sizes, shapes, and interior configurations. These are either commercially available or are manufactured in a laboratory. The next stage of development may be the structural model of theoretically anticipated nanostructures with the appropriate optical and physicochemical characteristics. An interesting strategy to bypass the functionalization stage is to utilize the functional components (drugs, peptides) as reducing agents.

Targeted delivery of anticancer medicines is a potential approach to the development of multi-functionalized GNPs in biomedicine. The objective was to improve the specificity of conjugate accumulation in tumors and to minimize the accumulation of conjugates in the reticulo-endothelial system. A vital step is to effectively target conjugates to malignancies.

Study reports indicate that cellular absorption effectiveness and the transportation of desirable substances into cells and tumors are influenced by size, shape, and surface coating and charge. The idea behind cancer immunotherapy is to stimulate the immune response of a patient to treat the tumor. New findings on effective clinical uses of cancer immunotherapy have highlighted the strategy’s enormous potential, particularly when combined with traditional physical and chemical remedies, medication schedule optimization, and accurate immune response monitoring. Cancer immunotherapy is still in its early stages, and more research is required to progress from the initially promising reported trials to FDA approval.

In particular, the combination of immune cells with empty or functionalized GNPs is yet to be completely defined and requires additional investigation. Nonetheless, the combined experimental results suggest that antigenic, adjuvant, and inflammation-inducing characteristics are significant components of GNP–immune system interactions. In this context, the rational design of GNP multifunctional nanoparticles could be essential to the ongoing growth of combined cancer therapies that incorporate the benefits of immunotherapy. The selection of an appropriate dosage and the design of therapy in early-stage clinical trials are significant issues. However, extensive research is required to generate biocompatible and stable nanoparticles that are capable of delivering a variety of medicinal drugs with enhanced pharmacokinetics, thereby resulting in superior cancer therapeutics and improved patient compliance.

## Figures and Tables

**Table 1:** FDA-approved immune checkpoint inhibitors.

FDA-Approved Drugs	Diseases
Ipilimumab (anti-CTLA-4)	Melanoma
Nivolumab (anti-PD1)	Multiple cancers
Pembolizumab (anti-PD1)	Multiple cancers
Atezolizumab (PD-L1 inhibitor)	Non-small cell lung cancer
Avelumab (anti-PDL1)	Merkel cell carcinoma
Durvalumab (anti-PDL1)	Bladder cancer

**Table 2:** Comparison between encapsulated and non-encapsulated compounds.

Encapsulated Compounds	Non-Encapsulated Compounds
Maintains drug concentration within therapeutic effective range. Provides optimum dose at right time and right location Effective use of expensive drug, excipients, and reduction in production cost.	This dosage form provides immediate drug release and can cause fluctuation of the drug level in the blood. Provides uneven drug distribution and leads to dose accumulation. Affects body’s normal cells in addition to targeted cells

**Table 3:** Advantages and limitations of GNPs in cancer treatment [98].

Advantages	Limitations
Biocompatible Facile synthesis Size and shape dependent optical properties Colloidal stability Intense absorption and scattering Increased molecule loading per particle Highly photothermal and photostable	Surface modifications alters pharmacokinetics, biodistribution, and toxicity profiles. Non-porous and non-biodegradable Limited penetration depth Reticuloendothelial system is affected

## Data Availability

Not applicable.
